# Development and Regeneration of the Zebrafish Maxillary Barbel: A Novel Study System for Vertebrate Tissue Growth and Repair

**DOI:** 10.1371/journal.pone.0008737

**Published:** 2010-01-15

**Authors:** Elizabeth E. LeClair, Jacek Topczewski

**Affiliations:** 1 Department of Biological Sciences, DePaul University, Chicago, Illinois, United States of America; 2 Department of Pediatrics/CMRC, Feinberg School of Medicine, Northwestern University, Chicago, Illinois, United States of America; Texas A&M University, United States of America

## Abstract

**Background:**

Barbels are integumentary sense organs found in fishes, reptiles and amphibians. The zebrafish, *Danio rerio*, develops paired nasal and maxillary barbels approximately one month post fertilization. Small in diameter and optically clear, these adult appendages offer a window on the development, maintenance and function of multiple cell types including skin cells, neural-crest derived pigment cells, circulatory vessels, taste buds and sensory nerves. Importantly, barbels in other otophysan fishes (*e.g.*, catfish) are known to regenerate; however, this capacity has not been tested in zebrafish.

**Methodology/Principal Findings:**

We describe the development of the maxillary barbel in a staged series of wild type and transgenic zebrafish using light microscopy, histology and immunohistochemistry. By imaging transgenic zebrafish containing fluorescently labeled endothelial cells *(Tg(fli1a:EGFP))*, we demonstrate that the barbel contains a long (∼2–3 mm) closed-end vessel that we interpret as a large lymphatic. The identity of this vessel was further supported by live imaging of the barbel circulation, extending recent descriptions of the lymphatic system in zebrafish. The maxillary barbel can be induced to regenerate by proximal amputation. After more than 750 experimental surgeries in which approximately 85% of the barbel's length was removed, we find that wound healing is complete within hours, followed by blastema formation (∼3 days), epithelial redifferentiation (3–5 days) and appendage elongation. Maximum regrowth occurs within 2 weeks of injury. Although superficially normal, the regenerates are shorter and thicker than the contralateral controls, have abnormally organized mesenchymal cells and extracellular matrix, and contain prominent connective tissue “stumps” at the plane of section—a mode of regeneration more typical of mammalian scarring than other zebrafish appendages. Finally, we show that the maxillary barbel can regenerate after repeated injury and also in senescent fish (>2 years old).

**Conclusions/Significance:**

Although the teleost barbel has no human analog, the cell types it contains are highly conserved. Thus “barbology” may be a useful system for studying epithelial-mesenchymal interactions, angiogenesis and lymphangiogenesis, neural pathfinding, wound healing, scar formation and other key processes in vertebrate physiology.

## Introduction

Development and regeneration are often studied in tandem, and share much in common. Development requires local coordination of cell division, distribution, differentiation, and death. Regeneration recapitulates these processes after injury or amputation, restoring some or all of the missing tissue. The ability to regenerate varies widely among species and organs in ways that defy simple evolutionary trends [1], [2], [3]. Within vertebrates, fishes and amphibians show the greatest regenerative potential [4], [5], [6], [7], [8], making zebrafish and *Xenopus* intensely studied models of this phenomenon.

Zebrafish tissues that can regenerate include the optic nerve, retina, heart, fins, lateral line, axons of the CNS and parts of the cerebellum [9], [10], [11], [12], [13], [14]. However, not every organ in this species has the same regenerative capacity. Solving this puzzle within the context of the zebrafish genome is an important step towards more effective regenerative medicine in highly refractory species, including our own. In this investigation, we explore the development and regeneration of the zebrafish maxillary barbel, an adult appendage that has received little research attention.

Anatomically, the term barbel refers to any tentacular sensory structure in “lower” vertebrates, including fishes, amphibians and reptiles [15], [16]. In fishes, barbels are skin appendages for taste and/or mechanoreception. Barbel number and construction are highly variable, with some species having up to 11 paired or unpaired barbels on multiple areas of the jaws, lips and head. Within a species, barbels can be sexually dimorphic or polymorphic among individuals of either sex [17]. According to anatomical descriptions, a teleost barbel contains at minimum an outer epithelium, dermal connective tissue, blood vessels, and extensions of the facial nerves that innervate numerous taste buds [16], [18], [19], [20], [21]. Variable features include a central rod of connective tissue or cartilage, and intrinsic and/or extrinsic muscle groups that allow the barbel some range of motion [22], [23], [24]. Although once used as a systematic character to unite all “fish with whiskers” (*e.g.*, the Barbini), barbels are now thought to be phylogenetically unreliable, having been gained or lost repeatedly in many genera, including *Danio* [25], [26], [27], [28], [29].

The zebrafish develops two pairs of barbels: a smaller nasal pair and a larger maxillary pair [30]. However, the most intensively studied part of the zebrafish lifecycle, namely the embryonic and early larval development of the first 7 days, does not include barbel growth. As a result, developmental information on these structures remains scant. A current search of the Zebrafish Information Network (ZFIN; http://zfin.org) retrieves no published or submitted gene expression data for the barbel primordium at any stage of development, no mutant/transgenic lines relating to this structure, and few related publications. One detailed study addresses the developmental distribution and innervation of barbel taste buds using light and scanning electron microscopy [31], but not cellular or molecular methods.

Abundant in the literature, however, are reports that some barbel structures can partially or completely regenerate after amputation. Studies of barbel regeneration in catfish are almost 100 years old, and various authors have periodically revisited this phenomenon [16], [32], [33], [34]. Sato (1966) removed the distal third of barbels from juvenile Japanese catfish (*Parasilurus asotus*) and observed healing of the wound after six hours, differentiating terminal taste buds after three days, and regrowth of the entire organ to the original size and length. Shiba (1982) removed the distal half of the barbel in bronze cory catfish (*Corydoras aeneus*) and observed a renewed appendage with taste bud structures in 2–3 weeks. More recently, barbel amputation was attempted as a mark-recapture technique for juvenile shortnose sturgeon (*Acipenser brevirostrum*); however, it was observed that many of the severed barbels completely or partially regrew, making this approach inappropriate for long-term studies [35], [36]. Although catfish and sturgeon barbels are not structurally identical to zebrafish barbels, nor are these structures necessarily homologous, these reports suggested to us that barbel regeneration might be evolutionarily conserved. Given the intensive study of other regenerating zebrafish organs– including the caudal fin, heart and eye– the absence of any experimental work on barbels seemed to us an obvious gap, and an opportunity to study simultaneously the developmental, evolutionary, and regenerative aspects of this unique appendage in a convenient model organism.

We begin by presenting a detailed study of the anatomy and histology of the adult zebrafish maxillary barbel. Next, we document development of the juvenile barbel from the early bud stage, considering the dermal connective tissues, taste buds, innervation and vasculature. Finally, we describe the regenerative response of the maxillary barbel to proximal amputation, including wound healing, blastema formation and re-differentiation of the major tissue types.

## Results

### Location and Growth of the Zebrafish Maxillary Barbel

**Figure 1:**
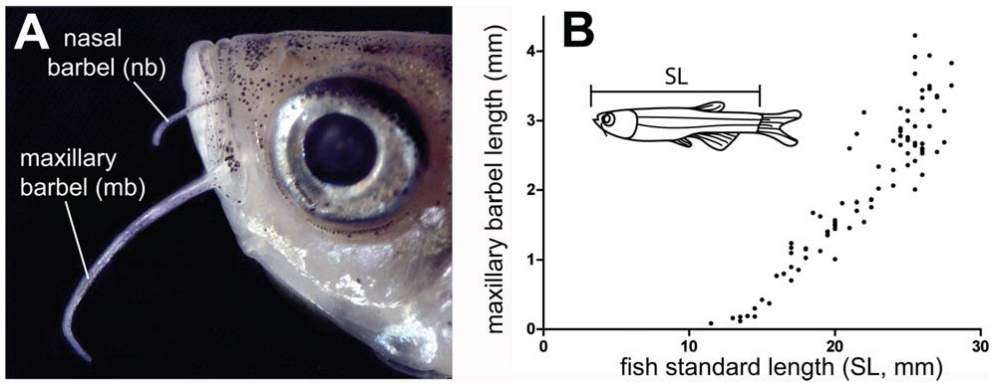
Position and growth of the paired barbels in zebrafish. **A)** Location of the nasal and maxillary barbels (**nb** and **mb**) on a wild type adult zebrafish (AB strain). **B)** Growth curve for the maxillary barbel in a wild type AB strain reared at 28°C. Barbel length (n = 183) was measured in 135 zebrafish of different standard lengths (SL+/−0.5 mm). Each data point represents a single barbel (the right and/or left appendage). The growth curve is similar to that shown in [60].

The maxillary barbel is an elongated whisker-like structure extending from the posterior ventral corner of the zebrafish maxilla (**Fig. 1A**). Both pairs emerge as epithelial buds approximately 30–40 days post-fertilization at 28°C [31] and grow throughout the lifespan (**Fig. 1B**). In adult wild type zebrafish in our facility (2–3 cm standard length, SL) the maxillary barbel is approximately 200–300 microns wide at the base, 50 microns wide at the tip, and 2–4 millimeters long. Although both nasal and maxillary barbels contain similar cell types, the larger maxillary barbel is easier to manipulate and visualize, making it the focus of our current study. By convention, we describe the maxillary barbel as though it were oriented horizontally with the distal end pointing caudally. In this orientation, the upper surface of the barbel is called dorsal and the lower surface ventral.

### Adult Barbel Anatomy and Histology

**Figure 2:**
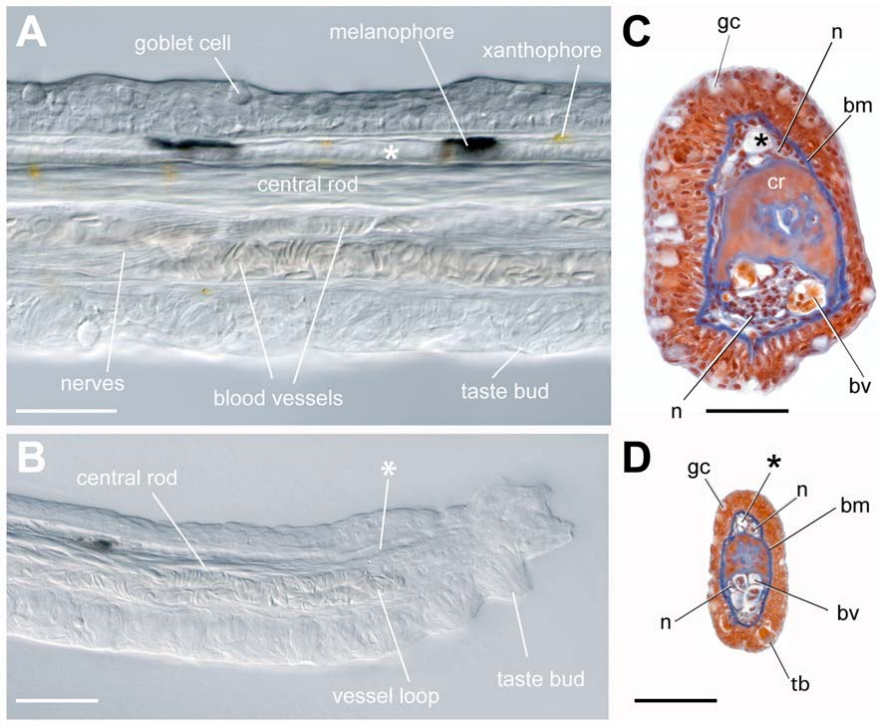
Whole-mount and sectional views of the adult maxillary barbel. **A)** Differential interference contrast (DIC) image of an adult maxillary barbel shaft fixed and cleared in 50% glycerol. All of the central tissues are visible through the transparent outer epithelium. A putative lymph vessel (*) lies dorsal to the central rod. All scale bars = 100 µm. **B)** DIC image of an adult maxillary barbel tip at the same scale as **A**. The central rod is reduced to a narrow band of fibers. The ventral vasculature terminates in a capillary loop packed with erythrocytes, while the lymphatic (*) terminates in a blind, tapered end. The ventral epithelium and distal tip carry numerous taste buds. **C)** Representative cross-section of an adult maxillary barbel at the level shown in **A**. Nuclei are dark red, erythrocytes orange, and basal laminae/connective tissues blue (Mallory's trichrome). **D)** Representative cross-section at the level shown in **B**. **bm** = basement membrane; **bv** = blood vessel; **gc** = goblet cell; **n** = nerve fibers; **tb** = taste bud; * = putative lymph vessel (for explanation see text).

Except for a few scattered melanophores and xanthophores, the adult maxillary barbel is transparent at all stages, making much of its internal structure visible with light microscopy alone (**Fig. 2A,B**). At the core of the barbel is a dense, refractile rod of connective tissue, hereafter called the central rod. Ventral to the rod are two small blood vessels closely packed with erythrocytes. The lumens of these vessels connect at the distal end of the appendage, establishing a capillary loop (**Fig. 2B**). Also ventral to the rod are large bundles of nerves visible as parallel fibers weaving in and around the blood supply (**Fig. 2A**). Dorsal to the central rod is a single, narrow vessel (see asterix in **Fig. 2A–D**), the lumen of which appears patent and filled with a clear fluid. This vessel has a blind, tapered end and does not appear to connect to the capillaries (**Fig. 2B**); thus, we tentatively identified it as a lymph vessel.

All of the deep barbel tissues– the central rod, nerve bundles and vasculature– are surrounded by a thick, glandular epidermis. Individual goblet cells and taste bud structures are easily observed. Under the dorsal epidermis there is typically a row of large, dark, and regularly spaced melanophores, though the arrangement of these cells is highly variable. The ventral epidermis generally lacks melanophores. Small, rounded xanthophores are scattered throughout the barbel epithelium; however, this pigmentation does not interfere with the structure's overall transparency.

To confirm the cellular features observed in whole mounts, adult maxillary barbels were embedded in paraffin, serially sectioned and stained with Alcian Blue/hematoxylin/eosin, Mallory's trichrome, or a modified Verhoeff's-van Gieson elastic stain. Mallory's stain proved the most informative for viewing the blood supply, as erythrocytes stain bright orange on a background of reds, blues and purples (**Fig. 2C,D**). For simplicity, only the Mallory's results are shown. In cross-section, the central rod was revealed to be largely acellular, lacking nuclei. This structure also had a slight dorsal-ventral asymmetry, with a convex dorsal side and a concave ventral side. In the middle of the rod there were often several small holes or voids, not noticed in whole-mount preparations, that contained solitary nucleated cells of uncertain histology. Some teleost barbels are known to contain a central rod of cartilage [18], [37]; however, the central rod in the maxillary barbel does not have a cartilage or bone histology, nor does it stain with Alcian Blue or Alizarin Red, two classic vertebrate skeletal stains (data not shown). This suggests that the rod is a non-mineralized extracellular matrix, most likely collagen, keratin or elastin. Whatever its composition, the central rod is not entirely homogeneous, as different areas show varying affinity for either the red (Orange G) or blue (aniline blue) components of Mallory's stain.

Ventral to the central rod we confirmed the two small capillaries (**Fig. 2C**). These were filled with orange-staining erythrocytes and lined with endothelial cells, which were identified by their elongated cell bodies and prominent nuclei bulging into the lumen of the vessel. A dense pad of myelinated nerve fibers surrounded both vessels. Dorsal to the central rod we observed the putative lymphatic. This vessel had a circular or oval cross-section and was also lined with endothelial cells. Smaller patches of nerve fibers, not easily visible in whole-mount preparations, surrounded this structure.

The epidermis of the maxillary barbel is a stratified cuboidal epithelium approximately 4–6 cells deep. At the barbel base this layer is approximately 40 microns thick, while at the distal tip it is 10 microns or less. This is because the individual epithelial cells at the tip are smaller (**Fig. 2C** *vs.* **2D**). Embedded within the epithelium are many large, Alcian Blue-positive goblet cells and, on the ventral surface, numerous taste bud structures with open apical pores. Mature barbels also have 10–12 spiny epidermal projections; in contrast to most areas of the teleost epidermis [38], these scattered projections appear keratinized (not shown). Finally, the entire epithelium rests on a thick basement membrane that stains intensely blue with Mallory's. Small breaks in this membrane were observed near the base of the each vase-shaped taste bud, through which fine nerve fibers projected to the sensory cells.

### Live Imaging of the Adult Maxillary Barbel Circulation

To test the lymphatic identity of the dorsal vessel observed within the maxillary barbel, we performed short-term live imaging of barbel blood flow in *Tg(fli1a:EGFP)* transgenic zebrafish. *Fli1a* (friend leukemia integration 1a) is a transcription factor constitutively expressed in the endothelial cells that line adult blood and lymph vessels [39], and is a frequent marker in studies of zebrafish angiogenesis and lymphangiogenesis [40], [41]. Under transmitted light (2–3 minutes/fish, n = 3), we observed a constant flow of erythrocytes along the barbel's ventral side, consistent with the location of the capillaries (**Movies S1** and **S3**). Erythrocytes flowed distally within one arm of the capillary loop, and returned proximally within the other arm. During the same period of observation, we saw no bulk flow of cells in the adjacent dorsal vessel, located deep to the dorsal row of melanophores. UV illumination of the same barbels, however, showed strong green fluorescence in all three vessels: the two capillaries ventrally and the “empty” vessel dorsally (**Movies S2** and **S4**). We infer from these preliminary observations that the dorsal vessel of the zebrafish maxillary barbel is both *fli1a:EGFP*-positive and blood-flow negative *in vivo*, a phenotype consistent with a lymphatic identity.

### Development of the Juvenile Maxillary Barbel

**Figure 3:**
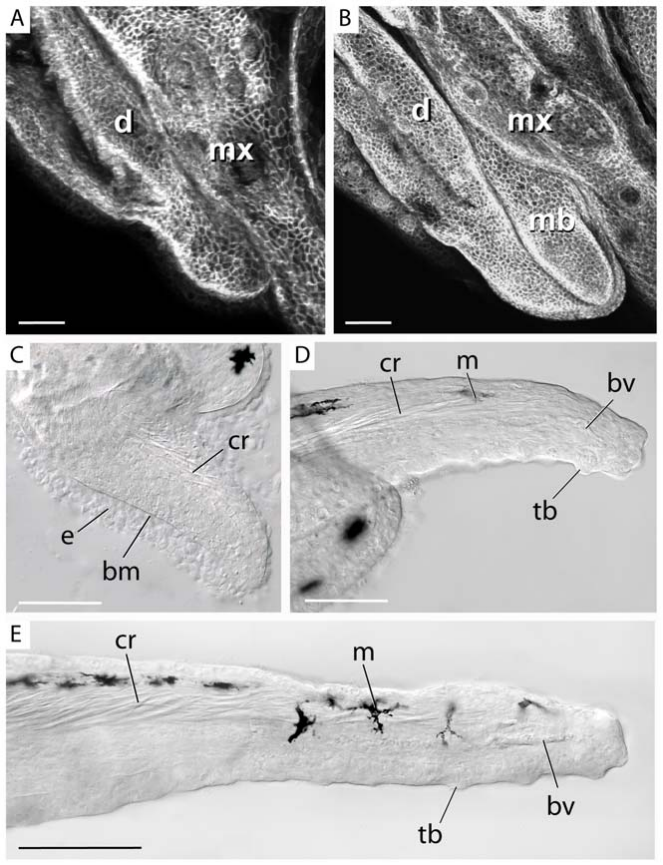
Early development of the maxillary barbel bud. **A)** Whole-mount confocal microscopy of the lower jaw of a juvenile membrane–GFP (mGFP) transgenic zebrafish (<10 mm standard length). Anterior is to the left. The maxillary barbel bud is not yet visible between the adjacent maxilla (**mx**) and dentary (**d**). All scale bars = 50 µm. **B)** Corresponding view of a slightly larger mGFP juvenile (10–12 mm SL). The first sign of the maxillary barbel (**mb**) is a rounded bud projecting caudoventrally. **C)** The early barbel bud has a thick outer epithelium (**e**) and a dense mesodermal core. The two layers are separated by a prominent basement membrane (**bm**). Fine strands of birefringent matrix accumulate dorsally where the central rod (**cr**) will form. **D)** As the barbel grows, the central rod becomes denser and projects farther distally. Isolated melanophores (**m**) appear along the dorsal epithelium. Ventrally, the blood vessel loop (**bv**) is forming. The ventral epithelium and distal tip carry numerous taste buds (**tb**). **E)** Later stages of barbel development involve expansion of the earlier structures. The central rod becomes longer and denser, the capillary loop extends, multiple melanophores become spaced along the dorsal surface, and numerous taste buds are added. The lymph vessel is not yet patent. **bm** = basement membrane; **bv** = blood vessel; **cr** = central rod; **d** = dentary; **e** = epithelium; **m** = melanophore; **mb** = maxillary barbel bud; **ms** = mesenchyme; **mx** = maxilla; **o** = orbit; **tb** = taste bud.

Having identified the major features of the adult maxillary barbel, we next investigated how this appendage develops. By 30–40 days post fertilization (>10–12.5 mm SL), the maxillary barbel primordium appears as a small raised bud projecting caudoventrally between the maxilla and dentary (**Fig. 3A,B**). Like a limb bud, the early barbel bud has two layers: an ectodermal jacket and a mesodermal core. At this stage the barbel is typically unpigmented, though the number of melanophores can vary from zero to two. Within the barbel core, many fine, refractile threads of matrix appear within the dorsal half of the appendage (**Fig. 3C**).

As the barbel bud matures (>12.5–15 mm SL; **Fig. 3D**), it changes from a rounded to a tapered outgrowth extending past the caudoventral margin of the maxilla. The refractile matrix in the mesodermal core becomes denser proximally and extends farther distally. Eventually, these matrix strands form a loose cone-shaped structure, wider at the base than at the tip, that corresponds to the position of the central rod seen in the adult. Ventral to the forming rod, angiogenesis is underway as indicated by the presence of endothelial cells in loose tubular aggregations. Two or more melanophores populate the dorsal epithelium. Multiple taste bud hillocks protrude from the ventral side and distal tip.

Later stages of barbel development (>15–17.5 mm SL; **Fig. 3E**) involve elongation and enlargement of the previous structures. The central rod becomes thicker and denser proximally, while the distal end resembles a loose mesh. Upwards of 10 melanophores are regularly spaced along the dorsal surface. A complete capillary loop is established, and overt blood flow occurs. The lumen of the dorsal vessel is still obscure, suggesting that this channel becomes patent somewhat later than the adjacent circulation.

### Development of the Maxillary Barbel Vasculature

**Figure 4:**
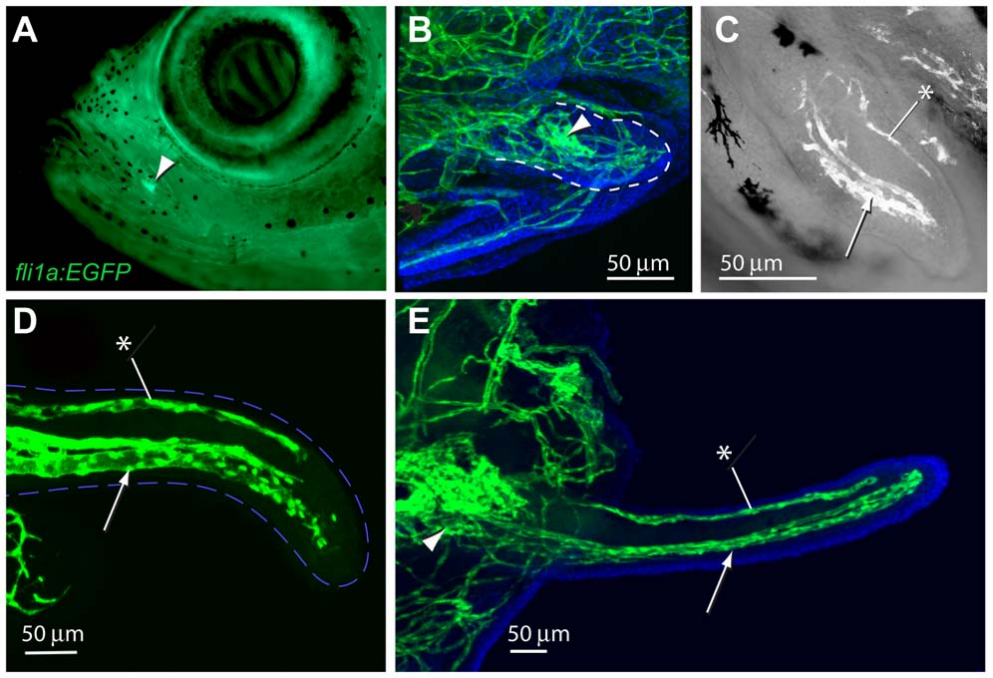
Development of the maxillary barbel vasculature in *Tg(fli1a:EGFP)* transgenic zebrafish. **A)** *In vivo* image of a juvenile *Tg(fli1a:EGFP)* zebrafish (10–12 mm standard length), in which all endothelial cells fluoresce green. The base of the future maxillary barbel is visible externally as a bright green confluence of blood vessels on the posterior ventral corner of the maxilla (arrowhead). **B)** 75 µm barbel. Confocal reconstruction of the early barbel bud circulation; anterior is to the left. A confluence of green vessels is visible at the base of the barbel (arrowhead). Smaller endothelial sprouts invade the bud proper (within the dotted line). Nuclei are counterstained blue (DAPI). **C)** 125 µm barbel. Two streams of endothelial cells are visible; a larger ventral stream (arrow), which will form the capillary loop, and a smaller dorsal stream (asterisk), which will form the putative lymphatic. In this focal plane, the proximal plexus of vessels is not visible. **D)** 300 µm barbel. The proximal ends of the ventral and dorsal vessels appear patent and lined with flattened endothelial cells. The distal ends of the vessels are composed of loose amoeboid cells with filipodia projecting into the surrounding tissue. The outline of the barbel is dashed blue. **E)** 600 µm barbel. The circulation at this stage consists of a closed capillary loop ventrally and a single, blind-end vessel dorsally. The proximal vascular plexus is greatly enlarged. Nuclei are counterstained blue (DAPI). Arrowhead = proximal vascular plexus; arrow = ventral vessels; asterisk = dorsal vessel (putative lymphatic).

To more closely examine the development of the barbel vasculature, we performed confocal microscopy on a developmental series of juvenile *Tg(fli1a:EGFP)* zebrafish collected 4–6 weeks post-fertilization (**Fig. 4**). In this transgenic line the approximate location of the maxillary barbel can be seen as a bright green dot on the juvenile lower jaw (**Fig. 4A**). This fluorescence is strong even before overt barbel outgrowth, and comes from a dense knot of endothelial cells immediately under the surface of the maxilla. We call this accumulation of vessels the proximal plexus, as it marks a persistent vascular plexus at the base, or proximal end, of the developing barbel. The plexus can be seen *in vivo* under a fluorescent dissecting microscope, providing a convenient pointer to the maxillary barbel's future location.

As the early barbel bud emerges, the proximal plexus projects 3–5 small endothelial sprouts distally, invading the mesodermal core (**Fig. 4B**). The pattern of sprouting was highly variable among individuals, and was often difficult to trace because of the density of vessels in this region. In the absence of dye- or cell-tracing studies, the connections between the proximal plexus and the barbel circulation are not well established. Based on its position, however, we assume that the proximal plexus is both the source of endothelial cells for the developing barbel and a likely reservoir for fluid flowing to and/or from the appendage.

By the stage of barbel growth beyond the margin of the maxilla, the endothelial cells within the barbel shaft are organized into two streams: a large ventral stream and a small dorsal stream (**Fig. 4C**). Consistent with the adult vasculature previously described, we infer that the ventral stream contains the capillary progenitors and the dorsal stream the lymphatic progenitors, respectively. The cells are not yet fully organized into tubes, and appear to be individually migrating through the surrounding tissue.

When the barbel has reached several hundred microns in length, the endothelial cells have formed several overt vessels, indicated by continuous tubes of flattened cell bodies with few spaces present between adjacent cells (**Fig. 4D**). The dorsal stream forms one narrow vessel, while the ventral streams form two closely apposed, larger vessels. As observed in the adult, the dorsal and ventral vessels appear separated along their entire length, Only at the distal end of the developing vessels did we observe widely spaced endothelial cells with prominent filipodia, suggesting active migration.

When the maxillary barbel is approximately one millimeter long, its vasculature is a smaller version of the adult organization (**Fig. 4E**). Both ventral vessels are tightly organized along their entire lengths, and their lumens are connected distally, forming a complete capillary loop. The dorsal vessel is also well defined and has a blind, closed end with its lumen separate from the adjacent capillaries. The mesodermal tissue around these vessels contains few or no solitary endothelial cells.

### Maxillary Barbel Innervation and Taste Buds

**Figure 5:**
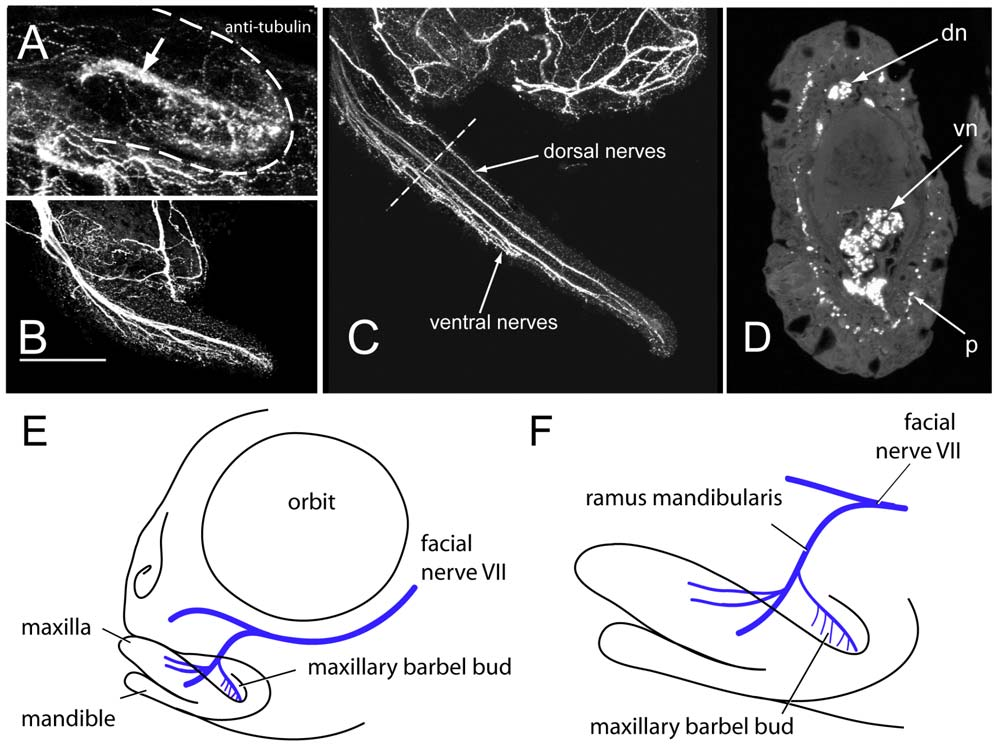
Innervation of the maxillary barbel. **A)** 75 µm barbel. In all panels, anterior is to the left. Whole-mount immunohistochemistry (anti-acetylated tubulin) shows a central tract of nerve fibers (arrow) within the early barbel bud (dotted white line). Smaller nerve projections are concentrated in the ventral half of the appendage. **B)** 200 µm barbel. Multiple fascicles of nerve fibers project distally, innervating the barbel's ventral side and distal tip. No large tracts are visible dorsally. Scale bar = 100 µm. **C)** 1 mm barbel. Secondary nerve fibers appear within the dorsal half of the barbel. **D)** Section of an adult barbel at the approximate level shown by the dotted line in C. Innervation is visible as two deep nerve tracts (**dn** and **vn**) and a ring of sub-epithelial immunoreactive punctae (**p**). **E,F)** Schematic reconstructions of maxillary barbel bud innervation based on confocal tracing of whole-mount acetylated tubulin immunostaining in multiple zebrafish juveniles. **F** is an enlargement of the jaw region in **E**.

As a taste organ, the maxillary barbel is well supplied with nerves. To trace the ontogeny of barbel innervation, we performed whole-mount immunohistochemistry using an antibody against acetylated tubulin [42], [43] to label the neurons in a developmental series of wild types. In juveniles of the smallest size class (>10–12.5 mm SL), a small tubulin-positive branch projects into the mesodermal core of the maxillary barbel bud, apparently innervating a ventral cluster of incipient taste buds (**Fig. 5A**). We also observed a nerve net of small, sinuous fibers throughout the barbel's epidermal sheath.

In the next size class (>12.5–15 mm SL), this pattern of barbel innervation is maintained and enlarged. The nerve trunk entering the barbel is thicker and brighter, and clearly composed of several roughly parallel fascicles (**Fig. 5B**). From these fascicles, dozens of smaller fibers descend ventrally, densely innervating the ventral epithelium. The epidermal nerve net appears more distinct and is evenly spread throughout the barbel surface. In the largest juveniles observed (>15–17.5 mm SL), the number of nerve fascicles in the barbel core increases (**Fig. 5C**); some fascicles stop short of the distal tip, while others extend the entire length. Also at this stage we observed a secondary innervation of the barbel, consisting of a large dorsal nerve fiber extending approximately half the length of the appendage. The origins and connections of this dorsal nerve are unclear.

Serial cross-sections of mature barbel tissue stained with the same antibody against anti-acetylated tubulin confirmed the pattern of innervation seen in whole mounts (**Fig. 5D**). In the adult barbel there are typically 1–2 dorsal nerve fascicles and 4–6 ventral ones. More fascicles are found in the thicker, proximal part of the barbel than at the narrow, distal tip. From these central nerve trunks we observed smaller fibers running radially towards the epithelial surface, penetrating gaps in the basement membrane and terminating near the bases of the vase-shaped taste buds. In sections, we saw scattered nerve fibers in the epithelium proper, but very bright signals from a sub-epithelial ring of immunoreactive punctae (**Fig. 5D**). Finally, by confocal imaging of small fish (<10–12 mm SL) stained in whole-mount, we were able to confirm the barbel's connection to the surrounding nerve supply (**Fig. 5E,F**). As previously described by other workers, a large trunk of cranial nerve VII, also called the facial nerve, descends from a trigeminal-facial nerve complex immediately ventral to the zebrafish orbit [44], [45]. This trunk sends a smaller branch, the ramus mandibularis, ventrally towards the jaw region. A secondary branch from this ramus innervates the maxillary barbel.

The early taste buds on the zebrafish larval head (3–5 dpf) and on the budding maxillary barbel have been previously described using light, scanning and transmission electron microscopy [31]. To examine the distribution of maxillary barbel taste buds in older juveniles and adults, we used whole-mount immunohistochemistry against the calcium-binding protein calretinin. This antibody labels a subset of differentiated taste bud cells in zebrafish, among other neuronal subtypes [46], [47], [48]. It also reacts with taste buds in other teleosts [49], [50], [51], [52].

**Figure 6:**
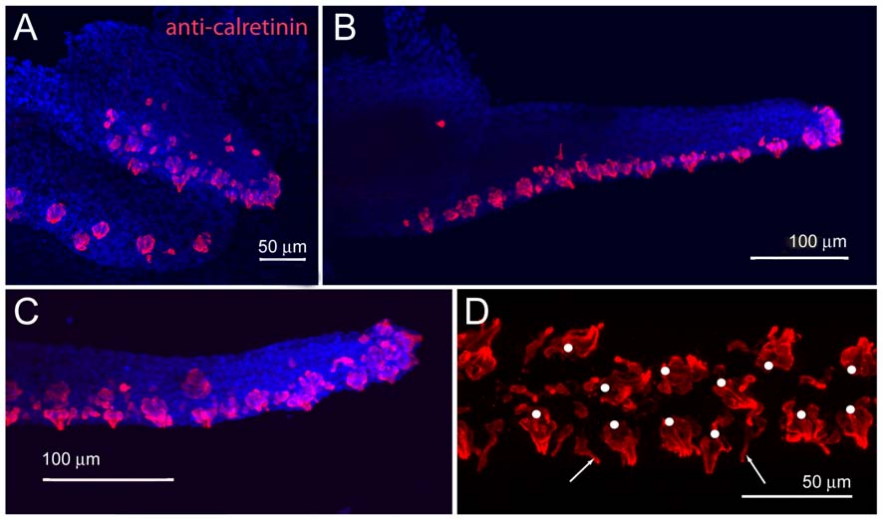
Maxillary barbel taste bud development. **A)** 150 µm barbel. Whole-mount immunohistochemistry (anti-calretinin) shows numerous differentiated taste buds (red) on the ventral surface and distal tip of the early barbel bud. Nuclei are counterstained blue (DAPI stain). **B)** 400 µm barbel. Teardrop-shaped clusters of calretinin positive cells line the ventral surface. **C)** Magnification of the maxillary barbel tip. **D)** Ventral view of the mature maxillary barbel. Teardrop-shaped taste buds (white dots) are arranged in pairs along the ventral surface. Scattered solitary chemosensory cells (SCCs, white arrows) are visible between the taste bud clusters.

The maxillary barbel bud appears well supplied with taste cells from the earliest stages of outgrowth, having numerous calretinin-positive cells on the ventral side and distal tip (**Fig. 6A**). As the barbel extends, these areas remain closely packed with onion-shaped clusters of immunoreactive cells (**Fig. 6B,C**); dorsally, few or no calretinin-positive cells are seen. In ventral view, the taste bud clusters of the maxillary barbel have a roughly paired arrangement (**Fig. 6D**), similar to the pattern of taste buds on catfish barbels [20]. At higher magnifications, two types of calretinin-positive cells are visible; cells arranged in clusters, but also the finger-like projections of solitary chemosensory cells (SCCs) [53].

### Maxillary Barbel Regeneration after Proximal Amputation

**Figure 7:**
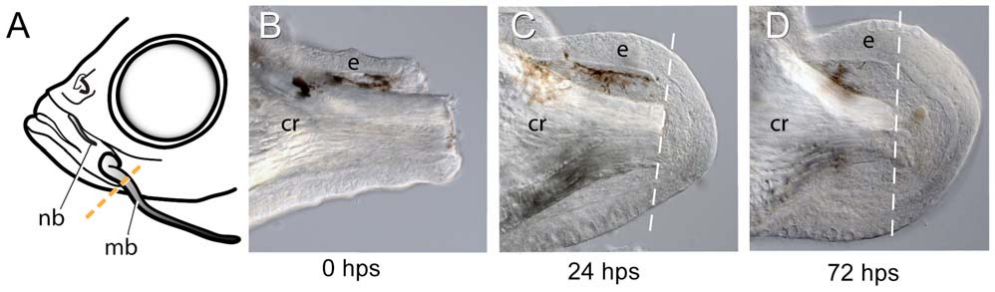
Proximal amputation of the maxillary barbel induces wound healing and blastema formation. **A)** Procedure for a unilateral “barbectomy”. The left maxillary barbel (**mb**) is amputated at the posterior margin of the maxilla. The contralateral barbel (not shown) is left as a non-surgical control. **B–D)** Fixed, unstained barbel regenerates collected immediately post surgery (**B**, 0 hps), or after 24 and 72 hours, respectively (**C**, **D**). Wound healing is followed by an accumulation of small, rounded mesenchymal cells underneath the epithelium (**e**) and around the central rod (**cr**).

Having established the normal morphology, histology, and development of the maxillary barbel, we next tested whether this organ could regenerate after surgical removal. We initially tried several sites of amputation, including the distal tip, mid-shaft and proximally near the base. All cut sites produced similar results (data not shown); however, because the proximal amputation produced the most dramatic regrowth and the greatest amount of regenerated tissue per fish, we eventually performed this operation exclusively. Another advantage of this location was that the amputation plane could be standardized by cutting the barbel shaft approximately where it crossed the margin of the maxilla, establishing an anatomical landmark for the original plane of section (**Fig. 7A**).

#### Wound healing and blastema formation in early maxillary barbel regenerates

Immediately after amputation, the barbel stump had a blunt end, exposing the deep tissues and central rod (**Fig. 7B**). 24 hours after surgery, this wound was well healed with a clear epithelial cap and an accumulation of small, rounded cells immediately underneath (**Fig. 7C**). Under the cap we occasionally observed small blood clots or patches of cloudy, presumably necrotic cells. These lesions cleared quickly and were not observed after the first few days. Blood flow in the severed capillaries was halted or irregular.

Seventy-two hours after surgery, the wound epithelium was thickened and the underlying mesodermal layer was enlarged, giving the distal end the profile of a slightly swollen bulb (**Fig. 7D**). A prominent basement membrane was reestablished, under which were numerous small, rounded mesenchymal cells forming a presumptive regeneration blastema. At this stage we observed the first differentiated cells in the regenerated portion– a few melanophores and xanthophores distal to the plane of section. Small capillary sprouts were carrying a few red blood cells into the swollen distal bulb, but a complete circulation was not yet established.

The early events of barbel regeneration were further examined by scanning electron microscopy (SEM) of the regenerating stumps. By collecting the regenerates at rather close intervals (0, 1, 3 and 6 hours post surgery), we hoped to capture “snapshots” of epithelial cell behavior during wound healing. At later time points (1, 3 and 7 days post surgery), we sought to establish when the regenerating maxillary barbel reestablishes its superficial sensory structures, particularly taste buds.

**Figure 8:**
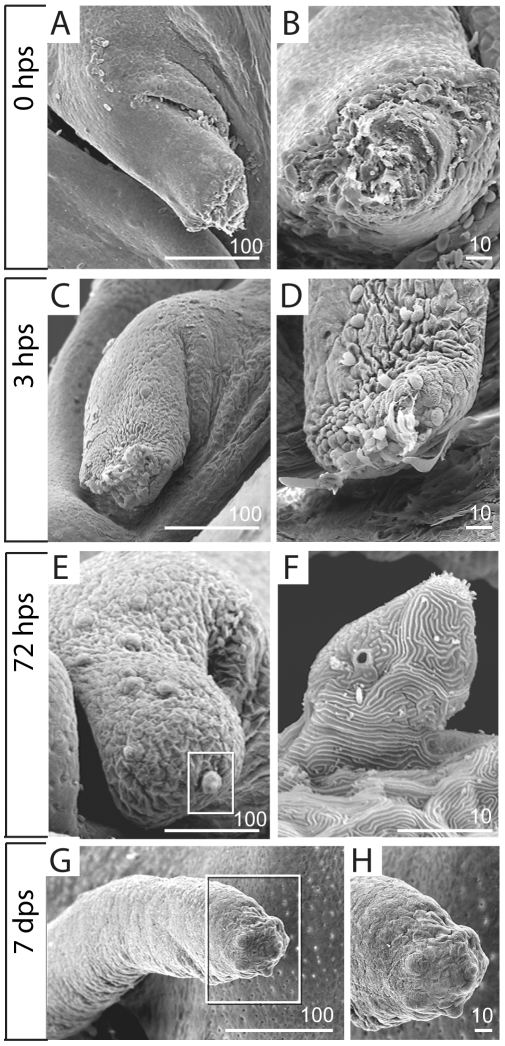
Scanning electron microscopy of early barbel regenerates. **A, B)** Immediately after amputation (zero hours post surgery, or 0 hps), the barbel stump is an open wound exposing the central core. **A)** Barbel stump in lateral view; proximal is to the left. **B)** End-on view of the same specimen. **C, D)** Two separate specimens collected at three hours post surgery (3 dps). The adjacent epithelium has closed the wound completely. Note the “purse string” lines of contraction within the epithelial sheet (**C**). Erythrocytes, dying cells, and matrix debris adhere to the distal surface (**D**). **E,F)** After 72 hours, the barbel stump swells distally, becoming bulbous (**E**). The tip epithelium carries newly differentiating taste bud hillocks, complete with protruding apical villi (**F**, magnified from **E**). **G,H)** By 7 days post surgery (7 dps), the maxillary barbel is a smaller version of the original appendage. Several millimeters long, it has a tapered distal end that carries a dense cluster of taste buds (**H**, magnified from **G**).

Barbels fixed immediately after surgery showed a ragged, cut surface exposing blood-filled sinuses and the central rod (**Fig. 8A,B**). After 3 hours, however, these wounds were completely closed although covered with dead or dying epithelial cells, scattered erythrocytes and cell debris (**Fig. 8C,D**). Sheets of skin appeared to converge medially and pucker over the wound, suggesting a “purse-string” action similar to that observed in SEMs of *Xenopus* wound closure [54]. One day after surgery, the distal surface of the regenerate was smooth and rounded, with no dead cells or surface debris (not shown). The length of the barbel was not increased, and the skin at the distal end showed no special epithelial characteristics. By 3 days post surgery the distal end of the barbel shaft became enlarged and bulbous, with several incipient taste bud hillocks at the distal tip (**Fig. 8E**). Each hillock was formed by the elevation of 3–4 epithelial cells topped by a crown of apical cilia (**Fig. 8F**). Seven days after surgery, regenerated barbels showed significant increases in length, having grown well past the original plane of section. Each carried at its distal end a cluster of 8–10 differentiated taste buds (**Fig. 8G,H**).

#### Later stages of maxillary barbel regeneration

By 7 days post surgery the regenerated barbels appeared to be smaller versions of the originals, although shorter and thicker than their contralateral controls. A complete capillary loop was re-established, with vigorous erythrocyte flow. A regularly spaced line of melanophores extended from the base into the regenerated region, approximating the original pigment pattern. At 14 and 28 days after surgery, the regenerated barbel was externally similar to a seven-day regenerate, but longer. Further growth was accompanied by progressive blood vessel elongation and melanophore migration, making the boundary between old and new tissue less distinct.

#### Proximal amputation of the maxillary barbel produces a permanent internal scar

**Figure 9:**
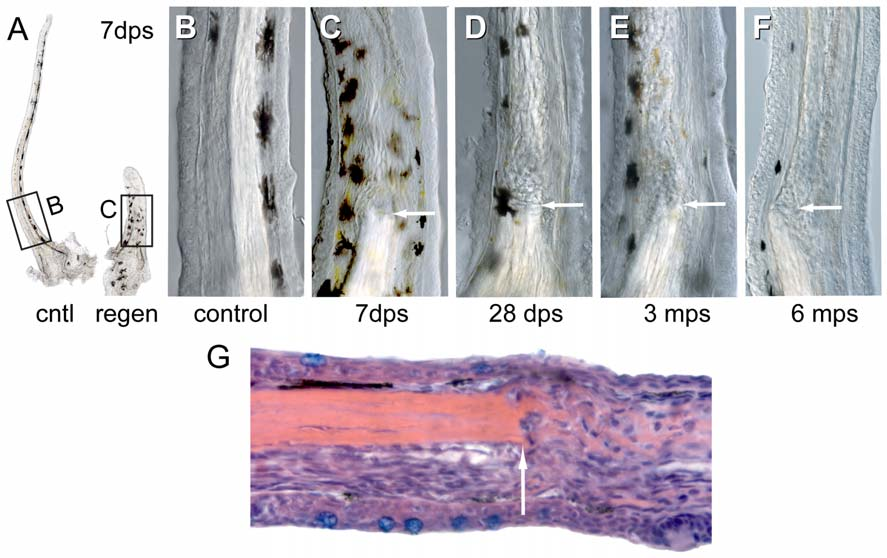
Internal scarring of maxillary barbel regenerates. **A)** Gross morphology of matched maxillary barbels (cntl = control; regen = regenerate) collected 7 days post surgery (dps). Note that the regenerate is thicker than the contralateral control and contains a central rod “stump.” **B)** Magnification of the control barbel in **A**. **C)** Magnification of the regenerated barbel in **A**. Note the absence of the central rod and the presence of wavy strands of matrix distal to the original amputation plane (arrow). The epithelial surface, pigment cell patterning and capillary loop are largely normal. **D–F)** Three regenerated barbels collected at 1–6 months post surgery (mps). All show disorganized mesodermal cores distal to the plane of section (arrow). **G)** Longitudinal histological section of a maxillary barbel regenerate (10 dps) showing disorganization of mesodermal cells and extracellular matrix distal to the amputation plane (arrow). Proximal is to the left. Hematoxylin/eosin stain.

Although the regenerated maxillary barbels appeared grossly normal *in vivo*, in fixed and cleared specimens we saw persistent differences between the regenerated appendages and their contralateral controls (**Fig. 9A**). Specifically, regenerated barbels were thicker and had abnormally organized mesodermal cores. We call this phenomenon the “internal scar” because the center of the regenerate, not the surface, is most affected. As the barbel regrows, the central rod is not replaced, but ends in a blunt stump within the epidermal sheath (**Fig. 9B,C**). Distal to the stump the barbel is filled with dense, wavy strands of birefringent matrix, intermixed with abundant nucleated cells (**Fig. 9G**). This morphology affects the entire shaft of the barbel distal to the amputation plane, extending several millimeters. The disordered matrix and scattered cells cause the core of the regenerates to appear “cloudy” under transmitted light, yet this does not interfere with confocal microscopy of the regenerated tissue.

We initially assumed that this dramatic scarring of the barbel would heal over time, and tested this by observing barbel regenerates and their contralateral controls at 1, 3 and 6 months after surgery. All of these older regenerates, however, had internal scars and matrix morphologies similar to those of recently operated specimens (**Fig. 9D–F**). We conclude that internal scarring of the zebrafish maxillary barbel is an acute and permanent reaction to amputation injury.

#### Regeneration of maxillary barbel vasculature, taste buds and nerves

**Figure 10:**
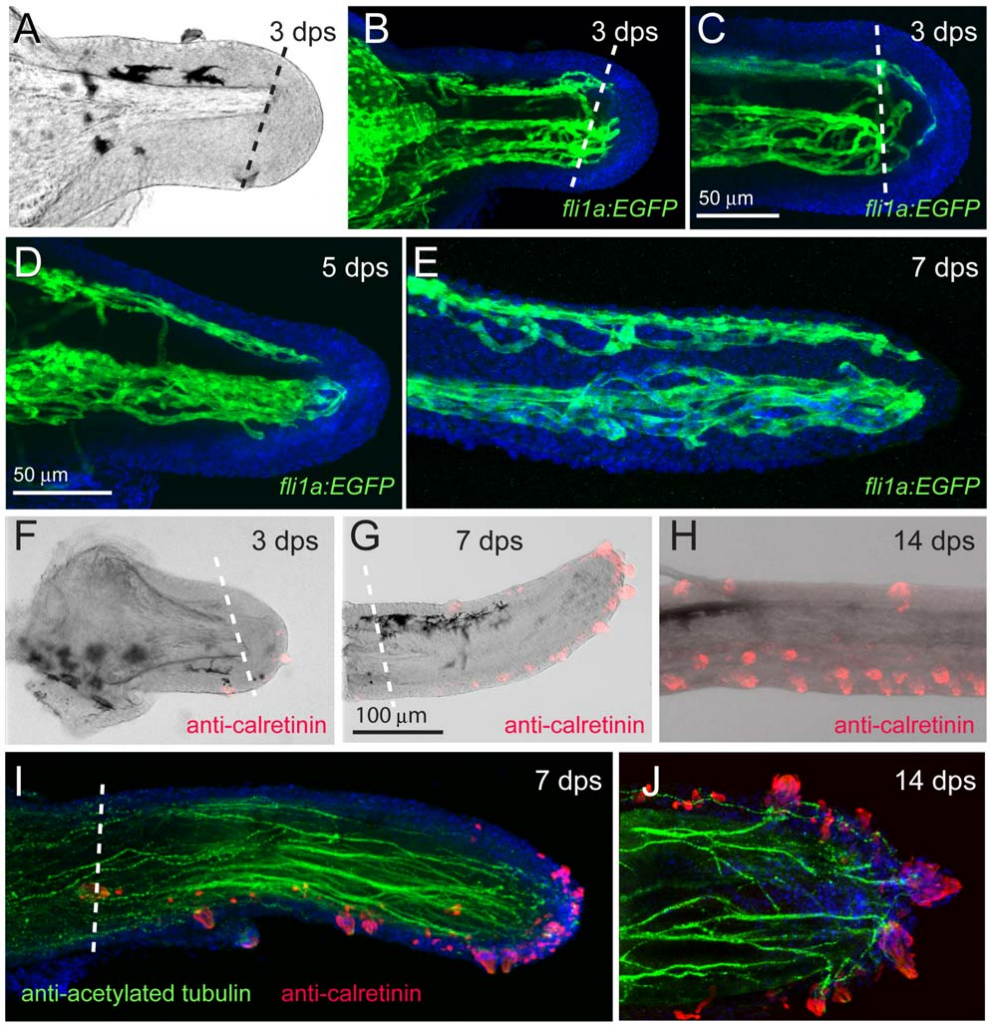
Regeneration of the barbel vasculature, taste buds, and sensory nerves. **A)** Transmitted light image of a *Tg(fli1a:EGFP)* maxillary barbel stump 3 days post surgery (dps). The distal end projects right. **B)** Confocal image of the vasculature in **A**. Endothelial sprouts (green) project distally past the plane of section and appear to bridge the dorsal (top) and ventral (bottom) vessels. Nuclei are counterstained blue (DAPI). **C)** Confocal reconstruction of a second regenerating barbel (3 dps). Similar sprouting is visible, as well as several isolated endothelial cells migrating underneath the wound epithelium. **D–E)** Regeneration of the vasculature at 5 and 7 days post surgery. Two streams of endothelial cells are visible; a dorsal stream (top) and a ventral stream (bottom). Both sets of vessels are more torturous than those of the original barbel (*e.g.*, **Fig. 4E**). **F–H)** Regeneration of the taste buds 3–14 days post surgery (dps). Calretinin-positive cells appear at the tip within 3 days (**F**). By 7 dps, the distribution of taste buds on the ventral side and distal tip resembles the normal adult pattern (**G**). The ventral taste buds of a 14-day regenerate (**H**) are arranged in a typical double row (compare to **Fig. 6D**). **I, J)** Regenerating maxillary barbels are densely innervated with long axons (anti-acetylated tubulin, green) projecting to the bases of the taste buds (anti-calretinin, red).

Given the disruption of the maxillary barbel's central matrix, we wondered how other barbel tissues would regenerate in this context. Specifically, would the barbel vasculature, taste buds and nerves be patterned normally in the regenerates, or would these be altered by the unusual extracellular environment? To examine the regeneration of the barbel vasculature in more detail, we performed an independent set of regeneration experiments on adult zebrafish strongly expressing the *fli1a:EGFP* transgene. Three days after surgery, there was no overt blood flow in the barbel blastema (**Fig. 10A**), but two sets of endothelial sprouts (dorsal and ventral) were observed in this area. These projected distal to the amputation plane and at times crossed dorso-ventrally across the central rod (**Fig. 10B,C**). Most sprouts appeared to follow the reformed basal lamina, migrating along the inner surface of the epithelium covering the wound. Within the blastema we also observed isolated GFP-positive cells with extended filipodia (**Fig. 10C**), similar to those observed in barbel development (**Fig. 4D**).

At later stages of regeneration (5–7 dps), dorsal and ventral vessels were extending distally, but with a modified morphology. Specifically, we observed larger diameters on some vessels, multiple vessels replacing single ones, and more bends and loops in the courses of the vessels. The single dorsal vessel, for example, might be replaced by two smaller, closely conjoined vessels or a complex series of anastomosing loops (**Fig. 10D,E**). The ventral vessels might be expanded from a simple capillary loop to a torturous set of conjoined lumens, all of which carried blood cells. The regenerated vasculature was thus highly variable; however, the dorsal and ventral populations remained separate, consistent with the hypothesis of distinct lymph and blood compartments. Barbels allowed to regenerate for longer than 7 days also had altered vessel morphologies, but did not depart from this basic regenerative pattern.

To examine the number, location, and pattern of regenerated taste buds, we stained barbel regenerates in whole-mount with an antibody against calretinin. Calretinin-positive cells were observed in the distal epidermis as early as 3 days post surgery (**Fig. 10F**), consistent with our scanning electron observations of the same interval (**Fig. 8E,F**). After one week, the distribution of taste buds on the regenerate was similar to the original, having large, differentiated buds concentrated on the ventral side and distal tip (**Fig. 10G**). The regenerated buds were arranged ventrally in a rough double row (**Fig. 10H**), restoring both taste bud patterning and appendage asymmetry. We also observed both types of calretinin-positive cells described previously, including onion-shaped taste bud clusters as well as solitary chemosensory cells (SCCs).

Finally, whole-mount immunostaining of barbel regenerates with an anti-acetylated tubulin antibody showed regrowth of a nerve supply very similar to that of the original appendage. Superficially, there were numerous small, torturous nerve fibers throughout the epidermis. Within the core, the regenerated portions of the barbel contained multiple axons projecting to the ventral side and distal tip, approaching the bases of newly formed taste buds (**Fig. 10I,J**). No taste buds were observed without nerve fibers, suggesting that the regenerated nerve fibers successfully found their sensory targets.

Although all of the regenerates accomplished this nervous rewiring, in some cases the locations of the central nerve trunks were abnormal. As previously described, a mature maxillary barbel has two principal regions of nerve fibers– a ventral region, adjacent to the capillaries, and a dorsal region, adjacent to the putative lymphatic (**Fig. 5D**). However, in several regenerates we observed only a single nerve tract ventrally, the dorsal fibers having shifted in this direction distal to the amputation plane (**Figure S1**). This outcome was rare, and its significance remains unclear. In general, we can conclude that the disorganized core of the maxillary barbel may alter, but does not preclude, the regrowth and repatterning of the deep tissues such as vasculature and nerves.

#### Measurements of regenerate growth

**Figure 11:**
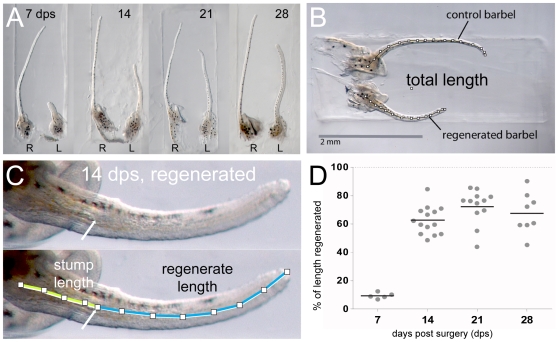
Barbel regeneration does not restore appendage length. **A)** Four matched pairs of maxillary barbels collected 7–28 days post surgery and embedded in the wells of a DNA electrophoresis gel. **R** = right barbel (unoperated control); **L** = left barbel (regenerate). All regenerates are shorter than the contralateral appendages. Each panel is shown at the same magnification. **B)** Measurement of total length (TL). The total length of each barbel (segmented line) was measured from the base of the central rod to the distal tip of the appendage. **C)** Measurement of stump length (SL) and regenerate length (RL). Within each regenerating barbel, the stump was measured from the base of the central rod to the amputation plane. The regenerate length was measured from the amputation plane to the distal tip. Stump length + regenerate length = total length of the regenerating barbel (SL + RL = TL). **D)** The regrowth of each regenerate was calculated as a percent of the control (% of length regenerated = (regenerate length/(control length – stump length))*100). Most lengthening occurred 7–14 days post surgery. Longer periods of regeneration (21–28 days) did not yield statistically significant differences in length.

In addition to their internal scarring and disorganized cores, regenerated maxillary barbels were consistently shorter than their contralateral controls. To measure this more precisely, we collected 40 matched pairs of barbels (regenerate and control) 1–4 weeks after surgery, embedded them in agar, and photographed them for digital morphometry (**Fig. 11A**). We measured the total length of each barbel (**Fig. 11B**) and, in the regenerated barbel, the length of the stump and the length of the portion that was regenerated (**Fig. 11C**). Assuming that both barbels had the same length initially, these measurements allowed us to express the regrowth of each regenerate as a percentage of the length of its contralateral control, allowing for differences in the stump length, which varied among surgeries. On average, the operated barbels were 2.9 millimeters long before surgery and 0.4 millimeters long after surgery, indicating that approximately 85% of the appendage was removed (**Table 1**).

**Table 1:** Barbel lengths pre- and post-amputation and during regeneration (7–28 days).

	mean value	median	range	standard deviation	sample size (n)
control barbel length (mm)	2.9	2.5	[2.2–2.8]	0.1	40
post-surgical stump length (mm)	0.4	0.4	[0.2–1.0]	0.1	40
percent length removed (%)	86.3	87.5	[72.2–92.7]	4.2	40
percent length regrown at:					
7 days post surgery (dps)	9.7	9.6	[7.1–12.7]	1.8	6
14 dps	62.7	61.0	[48.4–84.7]	9.1	14
21 dps	72.6	76.4	[44.0–85.6]	12.1	12
28 dps	67.8	66.6	[45.2–90.2]	14.4	8

Most regrowth occurred between 7 and 14 days, during which time the regenerates increased their average post-surgical length nearly sevenfold (**Fig. 11D**, **Table 1**). However the percent regenerated relative to controls was at 14 days highly variable (48–85%), with an average of just 62.7% of the missing length replaced. After 21 and 28 days this percentage was not significantly greater (72.6 *vs.* 67.8%, respectively) and the variability had not changed (45–85%). There was no effect of time on mean percent length regenerated for all comparisons among 14, 21 and 28 days (Kruskal-Wallis nonparametric ANOVA; H = 5.25, df = 2, p>0.07). We conclude that the maxillary barbel, although capable of restoring the majority of its missing tissue, does not replace its pre-surgical length. What halts the growth of the regenerate at this point is unknown.

### The Maxillary Barbel Can Regenerate after Repeated Amputation

Having established that the maxillary barbel could regenerate after an initial trauma, we wished to know if the regenerated tissue had any further regenerative capacity. Specifically, we sought to test if the disorganized cells filling the barbel core were “stem-like”, capable of further proliferation and differentiation, or “scar-like”, similar to pathological cells filling up a wound. We also wished to test if the presence of the barbel stump– specifically, the blunt end of the central rod– was necessary to organize a second round of regeneration.

78 adult fish (3–6 months post-fertilization) were anaesthetized and each had the left maxillary barbel removed. The regenerates were observed weekly to confirm that the first round of regeneration was typical. Approximately 4–6 weeks after the initial surgery, the first or “primary” regenerate was removed again, this time slightly distal to the first plane of section. The original, or primary amputation plane was located *in vivo* by illuminating the regenerate with strong transmitted light, making the internal scar more visible. The primary regenerate was fixed and stored while secondary regeneration was allowed to occur for an additional 4–6 weeks. At this point, all of the fish were euthanized to compare the primary and secondary regenerates with the unoperated controls using light microscopy with Nomarski illumination.

**Figure 12:**
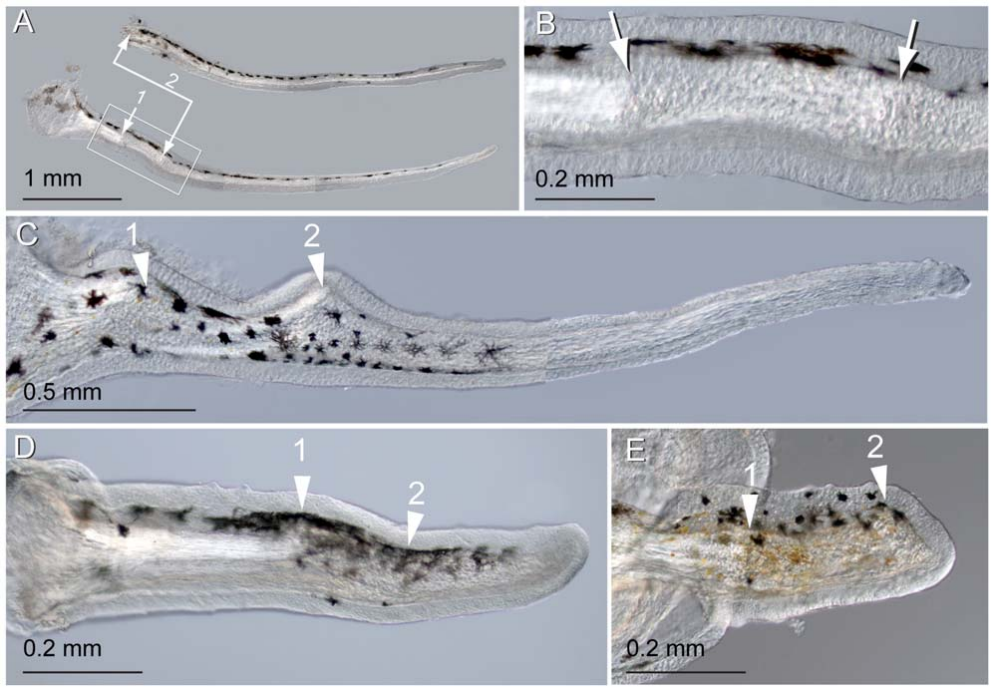
Repeated amputation can induce secondary regeneration. **A)** Two maxillary barbels regenerated from the same stump. The original barbel (not shown) was amputated at site 1 and the stump allowed to regenerate for one month. The resulting appendage (primary regenerate, top) was then amputated again slightly distal to the first amputation plane (site 2). A secondary regenerate (bottom) grew that was similar in size, shape and pigmentation to the primary regenerate. **B)** A magnification of the two surgical sites in **A**. Primary and secondary scars are visible approximately 0.5 mm apart. Note that the epithelial surface and melanophore patterning are largely normal. **C)** A secondary regenerate with more extreme scarring and swelling at the primary (1) and secondary (2) surgical sites. **D–E)** Failure of secondary regeneration. Secondary regenerates often failed to grow, elongating either slightly (**D**) or not at all (**E**) past the secondary surgical site (2).

Of the 78 fish challenged to undergo secondary regeneration, 66% (57/78) grew back a secondary maxillary barbel. Large secondary regenerates were patterned normally in terms of pigment cells, gross organization of the vasculature and taste bud distribution, and could be of comparable size to the primary regenerates (**Fig. 12A**). Notably, the internal scarring seen in primary regenerates was repeated in the secondary regenerates. In most cases, two separate scars were observed, one distal to the first (**Fig. 12 B,C**).

All of the secondary barbels deemed to regrow (n = 57) were scored semi-quantitatively for the amount of growth using values of 3 (growth of more than half the length of the contralateral control; **Fig. 12A–C**), 2 (growth >1 mm but less than half the length of the contralateral control; **Fig. 12D**) or 1 (growth <1mm; **Fig. 12E**). These secondary regeneration scores were strongly skewed towards “better” regeneration (score 3 = 32/57; score 2 = 18/57; score 1 = 7/57); however, the overall response was much worse than that of primary regeneration. Recall that in primary regeneration, 100% of regenerating barbels grew back 45–85% of the contralateral length (**Fig. 11D**). In secondary regeneration, less than half of the barbels grew more than half as long as the control appendage (32/78, or 41%), and nearly a quarter of the secondary surgeries induced no regenerative response at all (21/78, or 21%; **Fig. 12E**). We infer that, unlike the zebrafish caudal fin, the regenerative response of the maxillary barbel is diminished by repeated trauma.

### Maxillary Barbel Regeneration Occurs in Senescent Fish

Although most zebrafish in our study were 3–6 months old, we also tested the regenerative capacity of senescent fish over 2 years of age. 10 adult fish were anaesthetized and each had the left maxillary barbel removed. The fish were re-anaesthetized on the 3rd, 7th and 14th day after amputation to compare the gross morphology of the regenerate to that of the contralateral maxillary barbel. 80% of these fish responded with a normal progress of regeneration, completing extension and re-differentiation of the regenerate within two weeks. Although we did not examine the histology of these regenerates in detail, it appears that the ability of barbel tissue to regenerate, in most individuals, is life long.

## Discussion

In this report we have introduced the zebrafish maxillary barbel as a system for studying epithelial-mesenchymal development, wound repair, and regenerative cell biology. The maxillary barbel contains a simple cylindrical assemblage of ectodermal, mesodermal and neural crest derivatives, including skin, glands, pigment cells, taste buds, sensory neurons, blood vessels and a putative lymphatic. Barbels are optically clear, easily manipulated, and regenerate rapidly. In particular, the zebrafish system offers the ability to manipulate the genome, transcriptome and proteome of this unique appendage, which should prove beneficial in future studies of its molecular regulation.

### The Maxillary Barbel Contains a Putative Lymphatic

The zebrafish lymphatic system is of particular interest as its existence has only recently been described [41]. In the zebrafish embryo, lymph vessels are studied most commonly in the trunk or tail. However, these lymphatics are relatively short, deep, and often visually obscured by the adjacent segmental vasculature. In contrast, the maxillary barbel lymphatic has a straight path, a relatively large diameter, and can be several millimeters long. Assuming that this lymphatic compartment is further confirmed by molecular and functional assays, the maxillary barbel offers excellent visibility and access to isolated blood and lymph vessels in close proximity to each other. This arrangement could be used to study how these tissues coordinate their development and regrow into traumatized tissue after injury. Also intriguing is the ability of both vascular components to penetrate the complex mesodermal scar that forms after proximal amputation.

### The Maxillary Barbel Regenerates Some, but Not All Aspects of Its Original Structure

One definition of regeneration is the capacity to perfectly replace damaged structures. If the distal portion of the zebrafish caudal fin is severed, the appendage that regenerates is well integrated with the stump left behind. This includes both the internal fin rays (lepidotrichia) as well as the skin and pigmentation, making it a useful model of “perfect” or seamless integration. The zebrafish maxillary barbel, however, does not regenerate in this idealized way. Interestingly, it has an intermediate capacity for regeneration, achieving completeness in some tissues but not others.

The barbel is similar to the caudal fin in that its regenerative response to amputation is both consistent and rapid. At standard zebrafish rearing temperatures (28.5°C), overt regrowth ends approximately 2 weeks after injury. Barbel regenerates are grossly similar to the original appendages in both proportion and pigmentation. Histologically, the epithelial layer of the barbel is restored, including polysaccharide-secreting goblet cells and calretinin-positive taste buds in their expected anatomical locations. The vasculature and nerves also regrow, suggesting restoration of both circulatory and sensory physiology.

Unlike the caudal fin, however, the regenerated maxillary barbel fails to perfectly restore its original length or histological organization. The most striking difference between regenerated and control barbels is that the central rod of connective tissue is not replaced by a similar structure, but by an accumulation of rounded mesenchymal cells embedded within tangled strands of extracellular matrix– an arrangement resembling scar tissue. It was thus always possible to tell the regenerates apart from the controls, and to locate unambiguously the original plane of section. This internal disruption was correlated with abnormal pathfinding of endothelial cells and nerve axons, though these could still extend through the scarred tissue and establish grossly normal circulatory and sensory structures. Even after long periods of healing (3–6 months), the proximal parts of the regenerated barbels remained disorganized; however, the distal parts appeared more normal (not shown). We attribute this to new growth at the distal end of the appendage, as distinct from regeneration *per se*. However, as most of our anatomical observations are based on point samples, not longitudinal data, we cannot rule out morphological improvement in a single barbel over time through matrix remodeling, cellular turnover, or other long-term physiological processes.

Most currently studied vertebrate regeneration systems are remarkable for their rapid and largely seamless response to injury. Yet these systems are the least like our own physiology, in which the regenerative process is often tentative or aborted. This has been called the “experimental dilemma” of perfect regeneration [55]. Although perfectly regenerating systems can reveal in great detail how regeneration works, by definition they are less useful for testing therapeutic interventions. Thus we are faced with taking mechanisms gleaned from species that regenerate well (*e.g.*, fish and amphibians) and spanning a large phylogenetic gap to treat species that regenerate poorly (*e.g.*, most mammals).

If a zebrafish is capable of perfectly regenerating a severed tail fin, why not a severed maxillary barbel? Either barbel stump cells are not capable of the coordinated behaviors required for seamless integration, or they are capable of such behaviors but other constraints apply. It would be informative to discover, through a comparative study, what constraints are present in the barbel that are not present in the fin. The regenerating maxillary barbel might be developed as a therapeutic project within zebrafish, attempting to apply what is known about caudal fin regeneration to improve barbel “performance.” This represents a smaller, but still significant, biological gap to be crossed. For example, after amputation the barbel stump could be treated locally or systemically in ways that restore normal length and minimize scarring. In this way, the objective of manipulating the cellular environment from a more restrictive state to a more permissive one might be achieved.

### Origin and Replication of Cells in the Regenerate

A chronic problem in regeneration studies is to identify the source of cells making up the new tissue. New cells can come from the de-differentiation of surviving cells at the cut site, recruitment of pluripotent or “set-aside” cells previously contained within the stump, or recruitment of competent cells from distant parts of the organism. In the maxillary barbel, it seems likely that the epithelium regenerates by proliferation and expansion of the sheet that covers the wound. The nerves and vasculature presumably regrow by division and/or distal extension of surviving cells at the cut surfaces of these structures. The most unusual cells in the maxillary barbel are the disorganized matrix-secreting cells that replace the central rod. These are likely “fibroblast” or “stromal” cells of mesodermal origin. In the absence of lineage-tracing studies, however, a contribution from different or more distant cell types, perhaps entering through the capillaries, cannot be ruled out.

### Barbel Cells Are Pluripotent and Capable of Repeated Regrowth

A well-known feature of metazoan regeneration is that the differentiated tissues remaining at the injury site provide signaling molecules that direct the behavior of cells in the regenerate. In amphibian limb or zebrafish fin regeneration, this signaling is in part responsible for the seamless integration of the new growth with the old. The restored tissue, if amputated again, can repeatedly direct a new regenerate, allowing accurate regrowth even after several removals.

In regenerated maxillary barbels, the central core is dramatically reorganized. It contains more cells, more tangled strands of matrix, and, based on histological staining, perhaps an altered composition of matrix molecules. Secondary amputations through this core produced, in most cases, the regrowth of a secondary barbel similar to the first, or primary regenerate. We conclude that the regenerated central mesenchyme, although disorganized, can still support the growth of epithelial tissues and the distal extension of the vasculature and nerves. However, the regenerative response to the second round of injury was diminished, both in the number of individuals responding and the length of the structures produced. The reason for this variability is not known. Although the surgical technique was standardized as much as possible, it was more difficult to locate the secondary cut site in this operation. The distance between the primary and secondary cut site varied, which may have affected the results. We note that the variability of barbel regeneration after repeated injury is radically different than the response of the caudal fin, which can be removed many times and still replaces a full-sized, integrated tail. This is another way in which the caudal fin and barbel might be studied in parallel, to understand why one appendage retains and one loses regenerative capacity after repeated trauma.

### Fish with and without Whiskers: Development and Evolution of a Teleost Taste Organ

Although the mechanisms of barbel development and regeneration are most conveniently investigated in zebrafish, the study of these appendages has wider evolutionary implications for the Cyprinidae, the clade to which zebrafish belong. Historically, barbels have been used to classify cyprinid species into Linnaean groups; however, subsequent studies have discouraged this practice. Fox [16], citing the variable position and composition of teleost barbels, argued against any taxonomic usefulness or phylogenetic signal for these organs. Current trees for the family Cyprinidae show barbels evolving independently and repeatedly in many genera, including *Danio* [25], [26]. One ecological hypothesis for the frequent convergent evolution of this character is that barbel extension increases sensory sensitivity in low-visibility aquatic environments.

The demise of barbels as a systematic character is perhaps a gain for evolutionary developmental biology. Specifically, it opens up the study of the molecular factors controlling barbel placement and extension, and how these have evolved in various species of fishes. The fact that barbels have arisen independently in many cyprinids suggests that the mechanisms of barbel development are not so complex as to be rare; rather, like a switch, a barbel can be turned “on” or “off” rather frequently in evolutionary time. Assuming that the barbel develops by evolutionarily conserved epithelial-mesenchymal interactions, the initial signal for outgrowth is likely to come from one of a few gene families already well investigated in birds and mammals [56]. These signals could be tested in zebrafish juveniles. There also may be more than one way to build a barbel, in which case the underlying signaling pathways could themselves serve as characters in teleost phylogeny. Future investigations of these appendages might productively combine molecular biology, development, ecology and evolution in both barbeled and non-barbeled fishes.

### Potential Applications of the Maxillary Barbel System

In conclusion, the zebrafish maxillary barbel offers many of the advantages common to other vertebrate models of regeneration: speed, simplicity, transparency and easy access. Although the barbel has no human analog, the cell types it contains are critical for our development and maintenance, including skin, pigment cells, taste buds, nerves and vascular components. Most importantly, the techniques already available for zebrafish embryology and genetics can be immediately applied to this adult organ, which is similar to an embryo in shape and size.

Potential applications of the maxillary barbel system include studying the gene networks involved in skin appendage outgrowth and epithelial-mesenchymal responses to injury. The abnormal cell proliferation and matrix deposition that occurs during barbel regeneration may be relevant to vertebrate wound healing pathologies such as keloids or hypertrophic scars. The ability of barbel nerves to regrow to their taste bud targets may suggest approaches to restore peripheral nerve function to traumatized tissues. Finally, the availability within the barbel of both a capillary loop and a large lymphatic may be an attractive system to test the physiological and pathological functions of the adult circulation, including fluid balance and tumor metastasis.

## Materials and Methods

### Animal Care

All animal protocols were approved by the IACUC of Children's Memorial Research Center, (Chicago, IL) an AALAC-accredited facility. All strains were crossed and reared at 28.5°C using standard husbandry techniques. Five days post fertilization, larval fish were returned to flow-through tanks and fed a diet of powdered food and homogenized brine shrimp for two weeks. Surviving fry were then transferred to larger tanks at a density of not more than 1 fish/200 mL. All fish were fed live brine shrimp and commercial fish flakes twice daily for the remainder of the study.

### Strains, Developmental Staging and Sampling

For the wild type developmental series, we collected approximately 200 juveniles between 4 to 6 weeks post-fertilization from an inbred wild type AB strain (ZDB-GENO-960809-7; NU#1643). After fixation, the juvenile fish were sorted into three body size classes based on standard length (SL): >10–12.5 mm, >12.5–15 mm, and >15–17.5 mm. Of the several ichthyological definitions of standard length [57], we used the straight-line distance from the anteriormost point of the lower jaw to the base of the caudal fin (posterior limit of the hypural plate) measured to the nearest 0.5 mm. Fish <10 mm SL typically had no barbel outgrowths. Fish >17.5 mm SL had barbels that were longer than, but not structurally different from, the smaller size classes. Our developmental description is based on at least 30 individuals of each strain in each size class. Individual barbels were also “staged” during microscopy by measuring the length of the barbel from the proximal end of the central rod to the distal tip of the appendage. Because maxillary barbel length strongly correlates with fish standard length over a wide range of body sizes (**Fig. 1B**), either measure can be used to estimate developmental stage. For the examination of the vasculature in the *(Tg(fli1a:EGFP))* developmental series (ZDB-GENE-980526-426), we examined more than 100 selected larvae in the three size classes with at least 30 individuals per class.

### Paraffin Histology

Fish were euthanized in ice water and the desired tissues were fixed in cold 4% paraformaldehyde/phosphate-buffered saline (PF-PBS) overnight at 4°C. Fixed specimens were embedded in 2% agar and re-fixed in PF-PBS for 1–2 hours at room temperature. The agar blocks were then rinsed in PBS, dehydrated to absolute ethanol, cleared in Histoclear and embedded in Paraplast Xtra following standard histological schedules. At the 95% dehydration step, a brief wash in 1% alcoholic eosin:99% 95% ethanol stained the block light pink and the barbel tissue dark pink, improving visibility during later embedding. Wax sections were cut on a rotary microtome at 5–8 microns, briefly floated on warm (42–45°C) distilled water and mounted on Colorfrost Plus glass slides to dry overnight. For general histology we used an Alcian Blue/hematoxylin/eosin triple stain; for connective tissue, a modified Mallory's trichrome; and for nerves and elastic fibers a modified Verhoeff's-van Gieson elastic stain [58].

### Barbel Immunohistochemistry

The early barbel buds of juvenile zebrafish (approximately 10–15 mm SL) could be effectively stained as whole mounts. On the day of staining, freshly fixed tissues (PF-PBS for 2 hrs at room temperature or overnight at 4°C) were transferred to 1.5 mL Eppendorf tubes, adding no more than 100 µL of tissue volume per tube. Unless otherwise noted, each wash volume was 500 µL and the specimens were gently agitated at each step. After 5 washes in PBS+0.1% Triton-X (PBST, 5 min each), the barbels were permeabilized for 2–7 minutes in a 1∶10 dilution of 0.25% trypsin/2 mM EDTA (Mediatech, Inc. #25-053-CI) in PBST. Longer digestion times did not improve staining and pitted the epithelial surface. After 5 rinses in PBST, the barbels were blocked in 300 µL of PBST +10% goat serum for 1–2 hours.

Primary antibodies (mouse anti-acetylated tubulin; Sigma Chemical; rabbit anti-calretinin, Millipore) were diluted 1∶2,000 in PBST+1% goat serum. 300 µL of this solution was added to each tube for overnight incubation at 4°C. Control tissues were incubated in PBST+1% goat serum only. The next day, the barbels were washed in six changes of PBST (15 min each), followed by a 2-hour light-protected incubation at room temperature with a corresponding fluorescent secondary antibody (rabbit anti-mouse Cy3 or goat anti-rabbit Cy3; Jackson Labs) diluted 1∶200 in PBST+1% goat serum. After six more changes of PBST (15 min. each), the tissues were mounted in a 50∶50 solution of glycerol∶PBST and stored in the dark at 4°C until imaging. Double-labeling of nerves and taste buds in the same specimen was accomplished by first applying the anti-calretinin and detecting it with red, followed by an overnight wash, and then applying the anti-acetylated tubulin and detecting it with green (*e.g.*, goat anti-mouse FITC). If desired, DAPI was added to the mounting medium as a counterstain (final concentration = 0.0025 mg/mL). Brightfield and fluorescent images were captured on a Zeiss Axioscope and/or a Zeiss 510 META laser scanning confocal at 10–25× magnification.

To confirm that the staining patterns seen in whole-mounts of early barbels were valid, and to assure adequate penetration of antibodies into more mature barbel tissues, we repeated our immunostaining protocols on paraffin sections. Sections were prepared as above, dewaxed, rehydrated to PBST and processed without digestion using the same immunohistochemical solutions as described. After the final washes, the slides were coverslipped in 50% glycerol/PBS + DAPI and stored in the dark until imaging.

### Live Imaging of the Barbel Circulation

We crossed several pairs of *(Tg(fli1a:EGFP))* transgenic parents and screened the progeny at 3–4 dpf for strong GFP expression in the trunk and tail vasculature. Selected larvae were then raised to maturity (3–6 months). Most of the selected fish were used for the visualization experiments; others were set aside as founders for the next generation, with the goal of establishing a line with consistently high GFP expression in adult maxillary barbel endothelial cells.

For live imaging, each fish was deeply anaesthetized with buffered 0.015% Tricaine (MS-222, Sigma Chemical, pH 7.0) in system water and then placed right side up on a microscope slide. Two pieces of cellulose sponge glued to the slide held the midbody of the fish in gentle compression; the sponges were saturated with anesthetic solution and periodically rewetted throughout the procedure. The maxillary barbel was gently retracted from the lower (left) side of the head and extended over the glass surface. A drop of anesthetic water was placed on top of the barbel to keep it hydrated, and the barbel was brought into focus on the stage of a compound fluorescent microscope. Short video clips (10–30 seconds) of the barbel were recorded with differential interference contrast (for erythrocyte flow) and/or UV illumination (for the endothelially-expressed *fli1a:EGFP* marker). After 2–5 minutes, the fish was released to recover in system water. Video files were exported into Apple Quicktime Movie format and adjusted for brightness and contrast.

### Barbel Regeneration Experiments

To document the regeneration of barbel tissue, we surgically removed the maxillary barbel and observed the progress of the regenerates at various intervals. Our description is based on more than 750 repetitions of this procedure, each of which removed more than 85% of barbel tissue on the operated side. To minimize genetic variation, most of the fish in this part of the study were the offspring of random incrosses from 10–12 wild type AB parents. To control for the age and condition of the regenerating animals, most of the fish were raised at controlled densities (1 adult fish/200 mL water) on a standard lab diet and operated on between 3–6 months post fertilization. Males and females were reared in communal tanks, but never removed for crossing. The senescent fish (>2 yrs old) were adults from the same inbred line, but of different parents; their rearing densities and reproductive histories were unknown.

A video tutorial on maxillary barbel surgery and specimen collection is available at [59]. On the day of surgery, individual fish were lightly anesthetized in system water containing 0.015% buffered Tricaine. Each animal was transferred left lateral side up to a wet paper towel in a shallow Petri dish. Under magnification, a sterile fine forceps was used to elevate the left maxillary barbel, grasping it just distal to the posterior margin of the maxilla. A fine, sterile iris scissors was then inserted immediately proximal to the forceps to make a single cut roughly perpendicular to the barbel shaft. The right maxillary barbel was left unaltered as a control. After 2–5 minutes of recovery in fresh system water, fish were held overnight in an anti-infection tank containing 500 mL of system water plus one drop of methylene blue (Drs. Foster & Smith). The following morning, fish were returned to the rearing system. There were no complications and no fatalities. Tissues were collected for analysis either immediately after the surgery (0 hours post surgery = 0 hps) or at 1, 3, 6 or 24 hps. Later stages were collected at 3, 7, 14, 21 and 28 days post surgery (dps). Some fish were allowed to regenerate for even longer intervals, from 3–6 months. For regeneration experiments following the vasculature we used *Tg(fli1a:EGFP)* fish, at least 20 per time point, and collected the regenerates at 0, 1, 3, 7 and 14 dps.

### Scanning Electron Microscopy

Barbel tissues were fixed in 2.5% glutaraldehyde in 0.1 M sodium cacodylate buffer (Electron Microscopy Sciences, pH 7.4) at 4°C overnight. After 3 10-minute rinses in buffer, some specimens were then postfixed for 1 hour in 0.1 M sodium cacodylate containing 1% osmium tetroxide; others were exposed to buffer only. All specimens were then gradually dehydrated to 70% ethanol and stored at 4°C for several weeks. The day before imaging, the tissues were immersed in 80%, 90%, and 100% ethanol for 1 hour each, followed by 100% electron-microscopy grade ethanol overnight. On the day of imaging, specimens were either critical-point dried in liquid CO_2_ or air-dried on a paper towel. Once dry, all were mounted on stubs and sputter coated with gold. Images were obtained on the AMRAY 1810 scanning electron microscope at the Field Museum of Natural History in Chicago. Although all methods of tissue preparation produced acceptable results, postfixation in osmium followed by critical-point drying gave the best surface detail with the fewest artifacts.

### Linear Measurements of Developing and Regenerating Maxillary Barbels

For the developmental growth curve (**Fig. 1B**), maxillary barbels still attached to their maxillae were dissected from a series of 135 wild type zebrafish that had been previously measured for standard length (SL). From 48 specimens we collected both maxillary barbels and from 87 specimens we collected only the left barbel, for a total of 183 barbels measured. Each barbel was photographed next to a calibration scale in a Petri dish of buffered saline. Using the segmented line tool in ImageJ (http://rsbweb.nih.gov/ij/), we measured barbel length along the midline of the structure from the proximal end of the central rod to the distal tip of the barbel epithelium. Each barbel was graphed as a single data point.

For the measurement of regenerates and controls, matched pairs of maxillary barbels were rinsed in PBS and embedded in 2% DNA grade agarose/distilled water using a standard gel electrophoresis rig as an embedding mold [59]. Small-toothed combs were used to make shallow rectangular wells in the agarose. Under magnification, each pair of barbels was inserted into an empty well. The barbels were oriented parallel to each other with their proximal ends aligned. The moist agarose held the tissue in place by surface tension. After tissue positioning, excess fluid was removed from the well with a fine pipette tip or laboratory wipe. Fresh warm agarose was used to fill the well. A uniquely numbered paper label was inserted beside each well and covered with more agarose; for image calibration, a small plastic millimeter scale was embedded in the bottom of an empty well.

Once solidified, the gel slabs were wrapped in paper towels soaked in 1×PBS and stored in sealed plastic bags at 4°C until analyzed. Each pair of agar-embedded barbels was individually photographed on the stage of a dissecting microscope. The embedded calibration scale was photographed at the same magnification. Barbel lengths were measured in ImageJ using the segmented line tool to place points along the midline of the structure. For each pair of barbels, three measurements were taken: 1) total length (TL), from the proximal end of the central rod to the distal tip of the barbel epithelium (on control barbels), 2) stump length (SL), the distance from the proximal end of the central rod to the plane of section (on regenerating barbels only) and 3) post-surgical length (PSL), from the plane of section to the distal tip of the barbel epithelium (on regenerating barbels only). These measurements are diagrammed in **Fig. 11**.

To quantify the regenerative growth of the operated side, we made the simplifying assumption that the paired maxillary barbels were originally of the same total length. We further assumed, on the regenerating side, that all regrowth occurred distal to the amputation plane. Finally, we took into account the level of this plane, which varied, being slightly closer to or farther from the barbel base. Our calculation was thus (post-surgical length of the regenerate/(total length of the control - stump length of the regenerate)) = (PSL/(TL-SL)) = % length regrown. Thus, if the total length of the control barbel were 10 units and the regenerating barbel had been cut 1 unit from its base, we assumed that this regenerate had the potential to grow 9 units more, repairing 100% of the missing distance. If the total length of the control barbel were 10 units and the regenerating barbel had been cut 2 units from its base, this regenerate had the potential to grow 8 more units, also 100% of the missing distance. Calculations were performed in Microsoft Excel and exported to GraphPad Prism for graphing and statistical analysis.

## Supporting Information

Figure S1 Abnormal regrowth of maxillary barbel nerve tracts A) Whole-mount immunohistochemistry of a regenerated barbel (7 dps). Nerves = white (acetylated tubulin). The red star indicates the amputation plane. Proximal to this level (1), there are two major nerves tracts, dorsal and ventral. Distal to this level (2), there is only one. dn = dorsal nerves; vn = ventral nerves. B) Section of the specimen at level 2, showing all nerve tracts displaced ventrally (vn).
(5.49 MB EPS)

Movie S1 Blood flow in the shaft of a normal adult maxillary barbel (*fli1a*:EGFP transgenic line). The base (proximal end) of the barbel is to the left.
(1.71 MB MOV)

Movie S2 Blood flow in the tip of a normal adult zebrafish maxillary barbel (*fli1a*:EGFP transgenic line).
(2.56 MB MOV)

Movie S3 Fluorescent endothelial cells in the shaft of a normal adult zebrafish maxillary barbel (*fli1a*:EGFP transgenic line). The dorsal endothelial vessel that lacks blood flow (see Movie S1) is a putative lymphatic. The base (proximal end) of the barbel is to the left.
(2.39 MB MOV)

Movie S4 Fluorescent endothelial cells in the tip of a normal adult zebrafish maxillary barbel (*fli1a*:EGFP transgenic line). The narrow dorsal vessel that lacks blood flow (see Movie S2) is a putative lymphatic.
(2.27 MB MOV)
